# Ultrasonic Assessment of Females with Carpal Tunnel Syndrome Proved by Nerve Conduction Study

**DOI:** 10.1155/2013/754564

**Published:** 2013-06-20

**Authors:** Ihsan M. Ajeena, Raed H. Al-Saad, Ahmed Al-Mudhafar, Najah R. Hadi, Sawsan H. Al-Aridhy

**Affiliations:** ^1^Neurophysiology Unit, Physiology Department, Faculty of Medicine, Kufa University, Najaf, Iraq; ^2^Radiology Unit, Surgery Department, Faculty of Medicine, Kufa University, Najaf, Iraq; ^3^Pharmacology Department, Faculty of Medicine, Kufa University, Najaf, Iraq

## Abstract

*Introduction*. Carpal tunnel syndrome (CTS) is the most commonly diagnosed entrapment neuropathy of the upper extremity. The objective of this study was to diagnose CTS and to assess its severity using high resolution ultrasound (HRUS) depending on the results of nerve conduction study (NCS). *Methods*. A prospective cross-sectional study, in which HRUS was performed at 63 wrists of 35 female patients with different severity of CTS (as proved by NCS). Furthermore, 40 healthy volunteers (80 wrists) underwent the same tests as the patients and have been chosen to match the patients in gender, age, and body mass index (BMI). The cross section area (CSA) of the median nerve (MN) was obtained using HRUS at the carpal tunnel inlet by direct tracing method. * Results.* There was a significant difference in the CSA of the MN at the tunnel inlet in CTS patients when compared with the control group. In fact, the CSA of the control group showed a significant difference from each of patients subgroups. Furthermore, a significant difference in the CSA was seen in between these subgroups. In conclusion, the US examination of the MN seems to be a promising method in diagnosing and grading of carpal tunnel syndrome.

## 1. Introduction

Carpal tunnel syndrome (CTS) or compression neuropathy of the median nerve (MN) at the wrist is the most common form of peripheral entrapment neuropathy [1, 2]. It accounts for 90% of all entrapment neuropathies [3] and it is particularly prevalent in middle-aged women [4] and is recognized as one of the most important causes of the workplace morbidity [5]. The prevalence of CTS in the United Kingdom was 7–16% in 2010, while in the United States was only 5% [6]. The diagnosis of CTS involves combination of a detailed clinical history, accurate examination, and appropriate electrodiagnostic studies (EDS) [7].

High resolution ultrasound (HRUS) has emerged as a feasible, simple, relatively low-cost, rapid, accurate, and noninvasive imaging method for evaluating the MN in the carpal tunnel [1, 2, 4, 5, 8–21]. Despite that, some authors consider that the role of ultrasound scanning (US) in diagnosis of CTS is yet to be proven [22] and other stated that US appears to be of little use in the diagnosis of CTS [9]. By contrast, Wong et al. [10] proposed an algorithm involving initial US examination of patients suspected of having CTS and secondary EDS performed only when US results were negative. Furthermore, some studies stated that US could be used to grade the severity of CTS [18]. In addition to the detection of increased cross sectional area (CSA) of MN in patients with CTS, US may be used to detect space-occupying lesions as ganglia, fibromata, neural tumors, and tenosynovitis that usually cause CTS symptoms [9]. A previous prospective study compared the diagnostic utility of US versus EDS and found equivalent sensitivities between the two techniques [14]. Magnetic Resonance Imaging (MRI) has been shown to have a role where rare causes for CTS are suspected and also in the detailed reconstruction of the anatomy to aid endoscopic procedures [22]. Hence, the objective of this study is to diagnose CTS and assess its severity using HRUS depending on the results of nerve conduction study (NCS).

## 2. Materials and Methods

A prospective cross-sectional study was employed at Al-Sadir Medical City in Al-Najaf health directorate. Forty female patients with a provisional diagnosis unilateral or bilateral CTS were selected randomly from the outpatient clinic of the Teaching Hospital and the duration of their clinical symptoms ranged from 2 months to 15 years. Of these, only 72 hands showed positive NCS findings documenting the presence of CTS. Furthermore, nine hands of 5 patients were excluded later on during US examination due to the presence of anatomical variations in the MN or space occupying lesion. The remaining 63 hands fulfill the criteria of this study and were analysed as patient group. Forty healthy volunteers with no clinical signs or symptoms of CTS and normal NCS findings were included as control group. These volunteers had been chosen to match the patients in gender, age, and body mass index (BMI). All the participants had no history of upper limb trauma, no systemic diseases such as rheumatoid arthritis, diabetes mellitus, and thyroid dysfunction. Pregnancy and provisional diagnosis of cervical radiculopathy were other exclusion criteria. 

The study protocol was approved by the ethics committee of University of Kufa/Faculty of Medicine, and verbal consent was obtained from all patients and controls. 

For NCS, the subjects were examined in the Middle Euphrates Neurosciences Center, AL-Sadir Medical City using Electromyoneurography instrument (Micromed System plus-EMG, Italian model 2001). Patients were diagnosed to have CTS through NCS by testing the sensory and motor fibers of both median and ulnar nerves bilaterally with recorded median nerve abnormal conduction parameters. The results subdivided the patient group into three subgroups; mild (Grade 2 = only sensory fibers involvement), moderate (Grade 3 & 4 = in additional to grade 2, motor fibers involvement) and severe (Grade 5 & 6 = severe motor fibers involvement), according to the local severity scale of the neurophysiological reference values [23]. 

**Figure 1:**
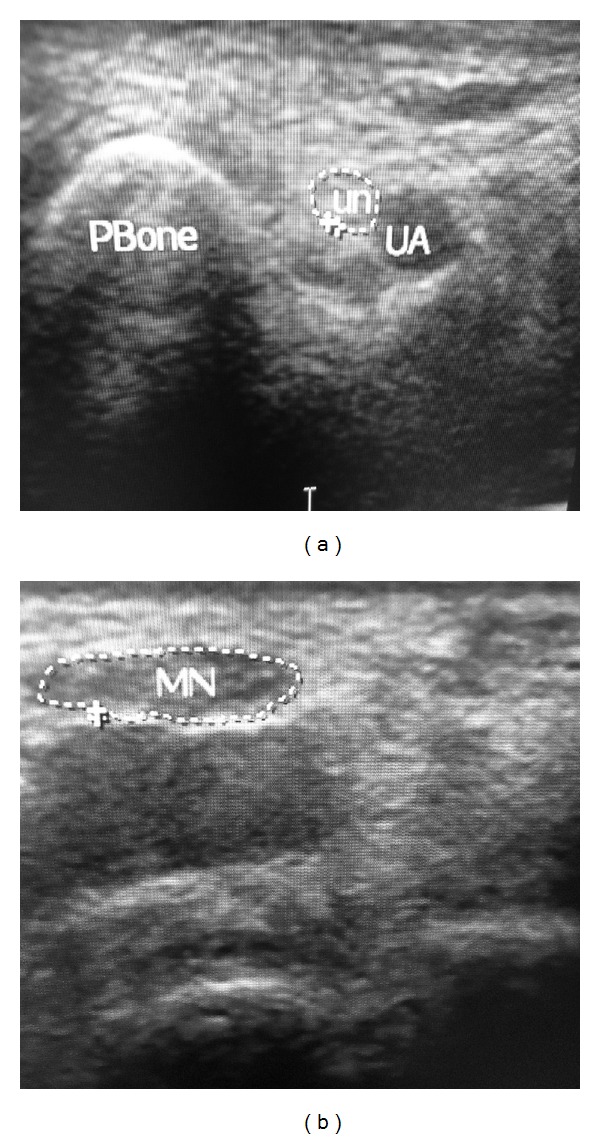
Wrist ultrasound at the carpal tunnel inlet for patient with mild CTS: (a) medial and (b) lateral aspects of the wrist (the tracing method for measuring the cross sectional area of the median nerve which was 10 mm^2^). UA: ulnar artery, UN: ulnar nerve in the Guyon canal, Pbone: pisiform bone, MN: median nerve.

**Figure 2:**
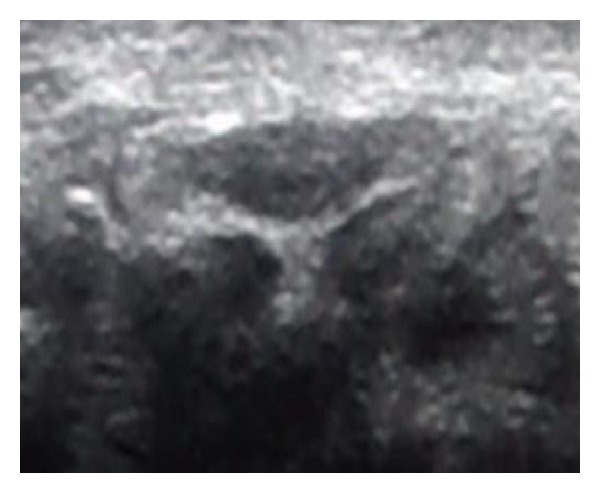
Wrist ultrasound at the carpal tunnel inlet for patient with moderate CTS, the cross sectional area of the median nerve was 14 mm^2^.

**Figure 3:**
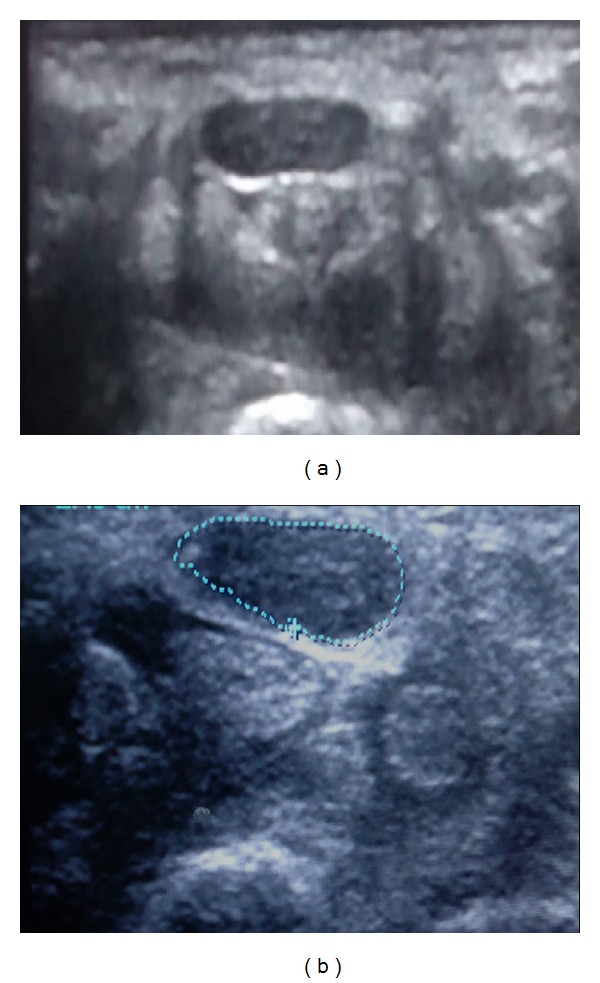
(a) Wrist ultrasound at the carpal tunnel inlet for patient with severe CTS medial, (b) the tracing method for measuring the cross sectional area of the median nerve which was 18 mm^2^.

The US examination was achieved using HD11XE Philips 2009 and the US unit equipped with a broadband 3–12 MHz linear transducer. The US evaluation was performed by the senior radiologist with special interest in musculoskeletal imaging who was blind to the degree of CTS severity reported by NCS at the time of the US study. Subjects were seated facing the examiner with their extended, supinated forearms, wrists were supported in a neutral position, and the fingers of that hand were semiextended. Ultrasonic gel was applied on the US probe to act as a coupling agent and then a real time transverse imaging of the MN from the distal forearm to the outlet of the carpal tunnel was performed. The CSA measurement of the MN was obtained using the standard protocol described by Duncan et al. [1] and Alemán et al. [24]. The technique of this protocol was as follows: (1) the transducer was positioned perpendicular to the MN, with no pressure on the skin to avoid deformation of the nerve; (2) axial images were obtained at the level of the pisiform bone, and the image with the optimal definition of the borders of the MN was selected; and (3) MN CSA measurements were performed from the inner border of the perineural echogenic rim, corresponding to the perineurium around the hypoechoic MN. Measurement was performed using the direct method, a direct tracing with electronic calipers around the margin of the nerve (Figures 1, 2, and 3).

Descriptive statistics of the measured CSA of the MN at the carpal tunnel inlet were presented as mean ± SD. Statistical analysis was carried out using Student's *t*-test and one-way ANOVA (Analysis of Variance) models to test differences between groups' means of continuous quantitative variables. The significance level (*P* value) *P* < 0.05 was considered as statistically significant. Data manipulation and analysis were performed using the Statistical Package of Social Sciences (SPSS) version 19 software.

## 3. The Results

The study population included 35 women with CTS as patient group and 40 volunteer women as control group. Both groups were matched in age and BMI (Table 1).

Only 28 (80%) of the 35 patients had bilateral CTS, while the remaining 7 patients (20%) had unilateral CTS. The dominant hand was affected in all of the unilateral cases. Of these 63 diseased wrists, 25 (40%) showed mild, 27 (43%) showed moderate, and 11 (17%) showed severe CTS according to electrophysiologic results.

The CSA of the MN at the tunnel inlet (at the level of the pisiform bone) in the patients group was significantly greater than that of the control group (Table 2).

There was a significant difference of CSA of MNs of different patient subgroups (mild, moderate, and severe) when compared to the control group (Table 3). Furthermore, a significant difference of this CSA was also noted between these subgroups when compared with each other (Table 4).

## 4. Discussion

In this study, a logical occasion that CTS present in mean age of (41.5 ± 6.5) years agrees with other researchers as Phalen [25] who reported that the peak age range of patients with CTS was 40–60 years [26] and Akcar et al. [4] who studied a sample with ages ranging between 33 and 58 years.

On comparing the BMI of participating patients (30.1 ± 4.8 kg/m^2^) with that of the control group (29.6 ± 3.7), the results showed no significant difference. It is known that a change in the BMI might affect the integrity of the nerve, a fact proved by many researchers as Werner et al. [27] who concluded that obese individuals (BMI > 29) are 2.5 times more likely to complain of CTS than slender individuals (BMI < 20). The correlation between CSA of the MN with BMI and hand physiognomies (small or strong wrists) may exist [9] and by 2012, Jessie et al. found that BMI had the greatest impact on ulnar nerve size [28]. That is why the control group of this study was selected to match the patients in regard to their BMI.

The participants were all females to avoid the effect of gender on results of the CSA as Andrea et al. recoded that CSA of the MN proximal to carpal tunnel was greater in men than in women by 2.2 mm^2^ [9].

The results revealed a significant increase of the CSA of MNs at the tunnel inlet in the patient group (13.11 ± 3 mm^2^) when compared with that of the control group (6.87 ± 1.04 mm^2^), a finding that is consistent with that of Akcar et al. [4]. At the same time, a significant difference in between three patients subgroups and with the control group was also recorded (*P* < 0.001).

It is not known exactly whether neuropathy of the MN develops as a result of intermittent mechanic compression or as a result of vascular compromise due to a rise in intracranial pressure [29], and perhaps both are responsible for the progression of CTS. Vascular compromise seems to occur in three stages: venous congestion; nerve edema, and impairment of the venous-arterial blood supplies [30]. A rise in pressure in carpal tunnel above normal, 20 to 30 mmHg, causes a chronic compressive ischemic injury to the nerve segment, resulting first in demyelination and eventually in axonal death. This will cause a progressive conduction block in the nerve with subsequent sensory and motor dysfunction [31]. When pressure builds on the median nerve, the blood supply to the outer covering of the nerve slows down and may even be cut off, causing ischemia. At first, only the outside covering of the nerve is affected but if the pressure keeps building up, the inside of the nerve will start to become thickened. New cells (fibroblasts) form within the nerve and create scar tissue [32]. 

The results of this study were in agreement with the results obtained by Karadag et al., who stated that the US was useful in grading the severity of CTS. They concluded that US measurement of CSA could give information about severity of MN involvement and they set US cut-off points that discriminate between different grades of CTS severity as follows: 10.0–13.0 mm^2^ for mild, 13.0–15.0 mm^2^ for moderate, and >15.0 mm^2^ for severe symptoms [18]. Also, El Miedany et al. [11] and Lee et al. [33] found that one can be confident of determining the level of severity of CTS based on US measurement of CSA of the MNs. In their work, they reported that US measurements of greater than 15 mm^2^ correlate with NCS findings of moderate to severe disease and noted that these figures differ significantly from those patients with mild to moderate disease. Furthermore, Moran et al. reported that the CSA of the MN at the tunnel inlet were 10.8 ± 1.9 mm^2^, 11.4 ± 1.8 mm^2^, and 12 ± 1.5 mm^2^ in patients with mild, moderate, and severe CTS, respectively. They reported that their clinical groups differed significantly from their control group (5.8 ± 0.9 mm^2^), but they found no differences between the patient groups [5]. This further underscores one of our principal findings. In even starker contrast to the present findings, Mohammadi et al. asserted that US cannot be used to grade the severity of CTS [8]. 

In the current study, the CAS of the MN was measured directly with electronic calipers around the margin of the nerve. This strategy has been employed in a number of studies that have reported that the direct method has greater diagnostic reliability than the indirect method (ellipsoid formula) [1, 2, 10, 11, 16].

Our results are consistent with previous reports that demonstrated the utility of US measurement of MN CSA at the tunnel inlet as a good alternative to NCS for the initial diagnosis of CTS [7, 10]. Furthermore, several other studies had concluded that CSA is the most predictive measurement for the diagnosis of CTS [2, 16, 17]. An interesting fact is that many studies showed the lack of interreader reliability of the CSA measurements obtained at the tunnel outlet [5] because MN may be difficult to be seen at outlet in persons with thick palmar skin and it has a wide variation as it usually splits into digital branches here [10]. That is why the current study used measurements of the CSA of the MN at the tunnel inlet despite the findings of Mohammadi et al., in 2009 about the usefulness of measuring CSA of the MN at the tunnel outlet [8].

In conclusion, the MN is easily visualized and measuring its CSA at the level of pisiform bone using HRUS is a sensitive, specific, and useful noninvasive method for the diagnosis of CTS. Furthermore, this diagnostic method is a reliable test in assessing the severity of CTS and might reveal some of its possible causes as space occupying lesion or anatomical variation of the MN. Finally, US examination of MNs seems to be a promising method for diagnosing and grading CTS.

## Figures and Tables

**Table 1:** The mean, standard deviation and range of age and BMI for the patient and control groups.

Sample	Number	Age/year		BMI (kg/m^2^)	
		Mean ± SD	Range	Mean ± SD	Range
Patients with CTS	35	41.5 ± 6.5	31–50	30.1 ± 4.8	23.5–38.1
Controls	40	37 ± 6.1	30–53	29.6 ± 3.7	23.4–36.6
*P* value	—	>0.05	—	>0.05	—

CTS: carpal tunnel syndrome, BMI: body mass index, SD: standard deviation.

**Table 2:** The descriptive statistics for CSA of MNs of patients and controls at the carpal tunnel inlet.

Sample	No. of wrists	CSA (mean ± SD)	95% Confidence interval for mean		Minimum	Maximum
			Lower bound	Upper bound		
Patients with CTS	63	13.11 ± 3.074	12.33	13.89	8.10	20.00
Control	80	6.87 ± 1.041	6.64	7.10	4.50	8.80
*P* value		<0.001				

CSA: cross sectional area of median nerve, CTS: carpal tunnel syndrome, SD: standard deviation.

**Table 3:** The CSA of MNs for the different patient subgroups versus that of the control group (depending on the results of the nerve conducting study).

CSA at the tunnel inlet (mm^2^)	Subgroups of CTS patients according to NCS			Control (*n* = 80)	*P* value
	Mild (*n* = 25)	Moderate (*n* = 27)	Severe (*n* = 11)		
	10.26 ± 0.83	13.81 ± 1.62	17.86 ± 1.89	6.87 ± 1.04	<0.001

CSA: cross sectional area of the median nerve, CTS: carpal tunnel syndrome, NCS: nerve conduction study.

**Table 4:** Multiple comparisons of CSA of the MNs of different patient subgroups with each other and with that of the control group.

Group type	Remaining groups	Mean difference	Significance
Mild CTS	Moderate	−3.55081	<0.001
	Severe	−7.59964	<0.001
	Control	3.39275	<0.001

Moderate CTS	Mild	3.55081	<0.001
	Severe	−4.04882	<0.001
	Control	6.94356	<0.001

Severe CTS	Mild	7.59964	<0.001
	Moderate	4.04882	<0.001
	Control	10.99239	<0.001

CSA: cross sectional area, MNs: median nerves, CTS: carpal tunnel syndrome.
